# Radiosensitization of clioquinol and zinc in human cancer cell lines

**DOI:** 10.1186/s12885-018-4264-2

**Published:** 2018-04-20

**Authors:** Shan Lu, Yuan Ke, Chaoyan Wu, Yahua Zhong, Conghua Xie, Yunfeng Zhou, Fuxiang Zhou, Haijun Yu

**Affiliations:** 1 Hubei Cancer Clinical Study Centre, Hubei Key Laboratory of Tumour Biological Behaviors, Wuhan, China; 2 Department of Radiation and Medical Oncology, Zhongnan Hospital of Wuhan University, Wuhan, China; 3 Hubei Cancer Clinical Study Centre, Zhongnan Hospital of Wuhan University, Wuhan, China; 4 Department of Integrated Traditional Chinese Medicine and Western Medicine, Zhongnan Hospital of Wuhan University, Wuhan, China

**Keywords:** Clioquinol, Zinc, Radiosensitization, NF-κB, DNA damage

## Abstract

**Background:**

We previously reported that clioquinol acts as a zinc ionophore and inhibits the NF-κB signalling pathway. Other research has demonstrated that zinc deficiency plays a vital role in the occurrence and development of some solid tumours, and intracellular zinc supplementation may reverse this process and enhance the tumour sensitivity to anticancer treatment. Thus, we investigated the radiosensitization effects of clioquinol combined with zinc on HeLa and MCF-7 cells in vitro.

**Methods:**

The dose effect of growth inhibition of clioquinol combined with zinc on cell viability was determined by a cell counting kit 8 (CCK-8) assay. The radiosensitization effect of clioquinol combined with zinc and/or MG132 in HeLa and MCF-7 cells was detected by the clonogenic assay. The cell cycle distribution and apoptosis of clioquinol combined with zinc on HeLa cells were analyzed by flow cytometry. A luciferase reporter construct was used to study the effect of clioquinol combined with zinc on NF-κB activity in HeLa cells. DNA double-strand breaks were detected by immunofluorescence. The mRNA and protein levels of ATM were analyzed by quantitative real-time PCR and Western blotting, respectively.

**Results:**

Our research showed that clioquinol combined with zinc markedly increased the radiosensitivity of HeLa and MCF-7 cells in low toxic concentrations and resulted in a post-irradiation decrease in G2 phase arrest and an increase in apoptosis. Clioquinol combined with zinc also inhibited NF-κB activation, decreased ATM expression and increased DNA double-strand breaks (DSBs) induced by ionizing radiation.

**Conclusions:**

These findings indicated that clioquinol combined with zinc enhanced the radiosensitivity of HeLa and MCF-7 cells by the down-regulation of ATM through the NF-κB signalling pathway.

## Background

Radiation therapy shows a significant clinical benefit in anticancer therapy. However, some solid tumours are resistant to radiation therapy, and the main reason is attributed to the radioresistance of individual cancer cells. Novel radiosensitizing agents can bring substantial benefit, demonstrating high radiosensitive efficiency and low cytotoxicity [1].

Zinc is a crucial trace element playing important roles in diverse physiological processes, such as growth, development, immune functions, and in the intracellular activity of numerous enzymes and transcription factors [2]. It is also effective in suppressing oxidative stress, clearing free radicals, sustaining genomic stability and decreasing the generation of inflammatory cytokines such as IL-1β and TNF-α [3]. Dysregulation of Zinc is associated with various pathological processes, especially tumour development [4].

Evolving and compelling evidence has shown that zinc is implicated as an important cytotoxic/tumour suppressor agent in several cancers. A recent meta-analysis showed that decreased zinc levels in serum and cancer tissue were found in patients with lung, head and neck, liver, stomach, and prostate cancers [5]. Serum zinc level assessment may improve cancer screening by identifying which patients should be subjected to further testing. Such procedures may increase the early detection of cancer. Thus, the zinc status may be a better indicator of the tumour burden and stage of disease than the overall nutritional status. Regulation of the intracellular zinc level will be a novel anticancer therapy. An increasing number of studies has indicated that zinc may be a significant radiosensitizing agent. First, zinc differentially modulates DNA damage in normal and cancer cells. Zinc protects normal cells against DNA damage, an effect that increases in tumour cells [6, 7]. Second, it has even been proposed that decreased serum zinc levels is a possible biomarker in cancer patients [5].

We have previously demonstrated that clioquinol (5-chloro-7-iodo-8-hydroxyquinoline, CQ) acts as a zinc ionophore and generates free zinc in lysosomes, leading to their disruption and apoptotic cell death [8]. Clioquinol (CQ), a derivative of chloroquine, has been used for many years as an inexpensive antifungal and antiparasitic agent and, subsequently, as a potential treatment for Alzheimer’s disease. More recently, it has been shown to induce cytotoxic effects in some malignant cells, acting as an ionophore with zinc-binding formation [9]. CQ has shown positive effects in the presence of zinc when combined with chemotherapy. To our knowledge, there are no reports concerning the effect of CQ and zinc on the radiosensitivity of tumour cells. If we can understand how they influence the response to DNA damage induced by ionizing radiation, it may be possible to use them more effectively in the clinical setting. In the present study, we aimed to investigate the radiosensitizing effects of CQ and zinc and their underlying mechanisms in vitro.

## Methods

### Reagents

Analytical grade clioquinol and zinc chloride were purchased from Sigma Chemical Co (Sigma Chemical Co, St. Louis, MO). CQ was dissolved in dimethylsulfoxide (DMSO, Solon, OH) to 10 mmol/L as stock solution (− 20 °C storage) and was diluted by MEM to a final concentration. Zinc chloride was dissolved in deionized water to 1 mmol/L as a stock solution (− 20 °C storage). The proteasome inhibitor MG132 was obtained from EMD Biosciences Inc. (San Diego, CA, USA). Antibodies against ATM, γ-H2AX, p65 and β-actin were obtained from Santa Cruz Biotechnology (Santa Cruz, CA, USA). The pNF-κB-Luc reporter construct was from BD Biosciences Clontech (Palo Alto, CA, USA), and the luciferase reporter assay kit was purchased from Promega (Madison, WI, USA).

### Cell culture and cell viability assay

HeLa (catalog number: ATCC®CCL-2™) and MCF-7 (ATCC®HTB-22™) cells were obtained from the American Type Culture Collection. Cells were cultivated in MEM (minimum essential medium) supplemented with 10% (*v*/v) FCS (foetal calf serum), 100 units/ml penicillin and 100 μg/ml streptomycin and were routinely grown in a 25-cm^2^ flask under a humid environment at 37 °C, 5% CO_2_. Cell viability was analyzed using a modified tetrazolium assay and a Cell Counting kit 8 (CCK-8, Beyotime, China) according to the manufacturer’s protocol. In brief, HeLa cells were plated in a 96-well tissue culture plate (2000 cells per well) with 100 μl of medium, which ensured 40–60% confluence after 24 h of growth. The medium was then replaced with 100 *μ*l of fresh medium containing CQ and zinc chloride (Sigma Chemical Co: St. Louis, MO) at various concentrations, and the cells were grown for designated periods. To each well, 10 *μ*l of the CCK-8 was added, and cells were incubated at 37 °C for 2 h to allow colour development. The plate was read at 450 nm, and the data were expressed as percentages of the values obtained from untreated control cells.

### Clonogenic assay

Cells were seeded into six-well plates at 100–2,000 cells/well depending on the dose of irradiation. Twenty-four h after seeding, cells were treated with DMSO, 5 μM CQ + 10 μM zinc, 50 μM MG132 + 5 μM CQ + 10 μM zinc (MG132 for 2 h prior to CQ and zinc), and then were exposed to various doses (0, 1, 2, 4, 6, 8 and 10 Gy) of γ radiation from linear accelerators (Primus High-Energy Siemens) at a dose rate of 2 Gy/min; a 1.5-cm bolus was used as a compensator, and the source skin distance (SSD) was 100 cm. After irradiation, cells were then grown for 10–14 days to allow for colony formation. Following that, cells were fixed and stained using crystal violet. Colonies with 50 or more cells were counted. The sensitizer enhancement ratio (SER) and other radiosensitization parameters were measured using GraphPad Prism 5.0 software according to the multi-target, single-hit model.

### Measurement of apoptosis

Cells were treated with CQ and zinc or DMSO for 4 h prior to treatment with 6 Gy of irradiation. Apoptosis was measured using propidium iodide (PI)/Annexin V-FITC double staining following the manufacturer’s instructions (BestBio Biotech, Shanghai, China) by method previously reported [10]. Cells were harvested 24 h after treatment with 6 Gy of irradiation, and the apoptotic fractions were measured using flow cytometry (Beckman, USA). Annexin-V+/PI- indicated early phase of apoptosis, while Annexin-V+/PI+ indicated late phase. The percentage of both cell types was counted. The data were expressed as the percentage of apoptotic cells per field.

### Cell cycle distribution analysis

Cells were treated with CQ and zinc or DMSO for 4 h, and then irradiated at 6 Gy. Twenty-four h after irradiation, cells were harvested and fixed with ethanol at 4 °C overnight. All samples were then washed with PBS and resuspended in PI (50 μg/mL) and RNase A (20 μg/mL) in PBS for 30 min at room temperature by a previously reported method [11]. Stained cells were analyzed by flow cytometry (Beckman, USA). The data were expressed as the percentages of different cell cycle distributions.

### Luciferase activity assay

Cells were grown in 100-mm dishes and were transfected with the NF-κB reporter construct (Palo Alto, CA, USA) using the Lipofectamine reagent (Invitrogen, Carlsbad, CA, USA), as previously described [9]. After 24 h of transfection, the cells were removed and plated into 96-well plates at 20,000/well. At 48 h of transfection, the cells were treated with 5 μM CQ and 10 μM zinc for 4 h prior to treatment with 2 Gy of irradiation. After 24 h of irradiation, the luciferase activity was assayed using the luciferase assay reagent. Briefly, the cells were lysed using reporter lysis buffer, and the insoluble material was removed by brief centrifugation. A total of 30 μl of luciferase assay reagent was mixed with 50 μl of protein extract, and the luciferase activity was analyzed using a Microplate Reader (Thermo Fisher Scientific, USA). The relative light units were normalized for the amount of protein in each extract, and the results were reported as percentages of the values obtained from untreated cells.

### Gama-H2AX foci detection

HeLa cells were seeded onto coverslips and were treated with 5 μM CQ and 10 μM zinc. After 4 h of co-incubation with CQ and zinc, the cells were irradiated with 4 Gy of γ-rays and were fixed, at intervals, in fresh 4% paraformaldehyde, 250 mM HEPES at pH 7.4, and 0.1% Triton X-100 for 20 min at 4 °C. After washing 3 times in ice-cold PBS, the cells were permeabilized in 0.5% Triton X-100 for 20 min on ice and then were washed and blocked with 5% FCS in PBS at 37 °C for 30 min. Coverslips were incubated with γH2AX antibody (Santa Cruz, CA, USA) overnight at 4 °C. After washing with PBS three times for 5 min, Alexa Fluor 488-conjugated anti-rabbit secondary antibody was added for 1 h at 37 °C, and the slides were washed with PBS and stained with PI. Finally, the images were recorded on a confocal microscope (Bio-Rad), and nuclear foci were counted in 20–30 cells per field [12, 13]. Each experiment was carried out in triplicate.

### RNA extraction and quantitative real-time PCR

MCF-7 cells were seeded onto 6-well plates and were treated with 5 μM CQ and 10 μM zinc. After 4 h of co-incubation with CQ and zinc, the cells were irradiated with 4 Gy of γ-rays. Twenty-four h later, total RNA was isolated from cell lines using TRIzol reagent (Invitrogen, USA) according to the manufacturer’s protocol. cDNA was synthesized from no more than 5 μg of total RNA using a PrimeScript@ First-Strand cDNA Synthesis Kit (Takara, Japan) at 37 °C for 15 min, 85 °C for 5 s, followed by 4 °C for 5 min. Real-time PCR was performed with SYBR Premix Ex Taq™ (Takara, Japan) in a 25-μL reaction volume (12.5 μL of SYBR Green Mix (2X), 0.5 μl of PCR forward primer, 0.5 μL of PCR reverse primer, 0.5 μl of ROX Reference Dye II (50×), 9 μL of ddH2O and 2 μL of cDNA template) using an MJ Opticon Monitor Chromo4™ instrument (Bio-Rad, CA). The human GAPDH gene was evaluated as an internal control. The following protocol was used for GAPDH and ATM (Santa Cruz, CA, USA): preincubation at 95 °C for 7 min, followed by 30 cycles of 95 °C for 30 s, 56 °C for 35 s, 72 °C for 30 s, and 72 °C for 10 min. Sangon Biotech (Shanghai, China) assisted in the design and synthesis of the primers for ATM and GAPDH as follows: ATM (forward primer 5’-CCTACCAAATCCCTCCACC-3′, reverse primer 5’-CCTTGAGCATCCCTTGTGTT-3′); GAPDH (forward primer 5’-TGGAAGGACTCATGACCACA-3′, reverse primer 5’-TTCAGCTCAGGGATGACCTT-3′). The data were analyzed by the 2-DDCt method.

### Western blotting

Western blotting was performed as previously described [14]. Briefly, HeLa cells were treated with CQ and zinc for 4 h prior to treatment with 4 Gy of γ irradiation and were lysed at intervals in a lysis buffer containing 50 mM Tris/HCl (pH 7.4), 100 mM NaCl, 5 mM Na/EDTA, 1 mM PMSF, 0.1% SDS, 1% (*v*/v) Triton X-100 and 2% (v/v) glycerol. The lysates were separated by SDS/PAGE (15% gels), transferred to a PVDF membrane, and blotted with antibodies against ATM, p65, γ-H2AX and β-actin.

### Statistical analysis

The data were analyzed with GraphPad Prism 5.0 (GraphPad Software Inc., USA), using t-tests to determine differences between two groups and one-way analysis of variance (ANOVA) for three or more groups; *p* < 0.05 indicated statistical significance.

## Results

### Clioquinol combined with zinc induces cytotoxicity in HeLa cells

**Fig. 1:**
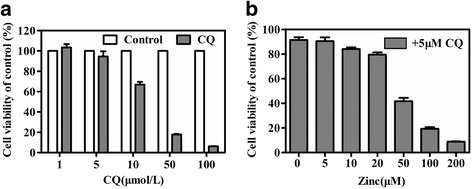
Effects of CQ and/or zinc on the viability of HeLa cells. CQ (**a**) or CQ combined with zinc (**b**) induced cytotoxicity in HeLa cells. Cells were exposed to the indicated concentrations of CQ or CQ combined with zinc for 72 h. Cell cytotoxicity was assessed by the CCK-8 assay. The data are shown as means ± SEM of three independent experiments

To evaluate the anticancer effect of CQ and zinc on cancer cells, HeLa cells were treated with different concentrations of CQ and/or zinc. The CCK-8 assay revealed that the inhibitory effects elicited by CQ and/or zinc were dose dependent (Fig. 1). To evaluate the effect of CQ and zinc on the radiation sensitization of tumour cells, the 5 μM CQ + 10 μM zinc group that induced approximately 15% inhibition of HeLa cell viability was selected for subsequent experiments.

### Clioquinol combined with zinc increases the radiosensitivity of cancer cells

**Fig. 2:**
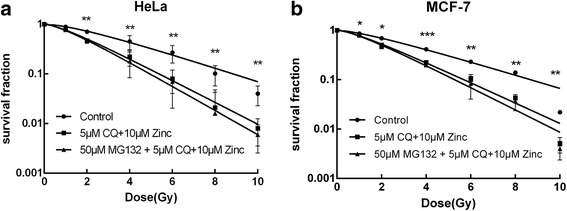
Effects of CQ and zinc on the radiosensitivity of cancer cells. Clonogenic cell survival curves were generated for HeLa (**a**) and MCF-7 (**b**) cells that were treated with DMSO、5 μM CQ + 10 μM zinc、50 μM MG132 + 5 μM CQ + 10 μM zinc (MG132 for 2 h prior to CQ and zinc), and then were exposed to various doses (0, 1, 2, 4, 6, 8 and 10 Gy) of irradiation. The survival data were normalized to those of the unirradiated control group. The data are shown as the means ± SEM for three independent experiments. **P < 0.01

To investigate the influence of CQ and zinc on the radiosensitivity of cancer cells, we performed a clonogenic cell survival assay. Dose-survival curves were plotted according to the multi-target single-hit model (Fig. 2). Cells pretreated with 5 μM CQ and 10 μM zinc plus γ-ray irradiation exhibited significantly lower clonogenic survival fractions at each dose than cells treated with radiation alone. The sensitizer enhancement ratios (SERs) were 1.55 and 1.53 in HeLa cells and MCF-7 cells, respectively. Pretreatment with MG132 (50 μmol/L for 2 h prior to CQ and zinc) enhanced the radiosensitivity in both cell lines, and the difference in the surviving fraction values compared with the 5 μM CQ + 10 μM zinc group was not statistically significant (*p* > 0.05). Our results indicated that treatment with CQ combined with zinc-sensitized cancer cells to γ-ray irradiation through the NF-κB signalling pathway.

### Clioquinol combined with zinc induces apoptosis in HeLa cells

**Fig. 3:**
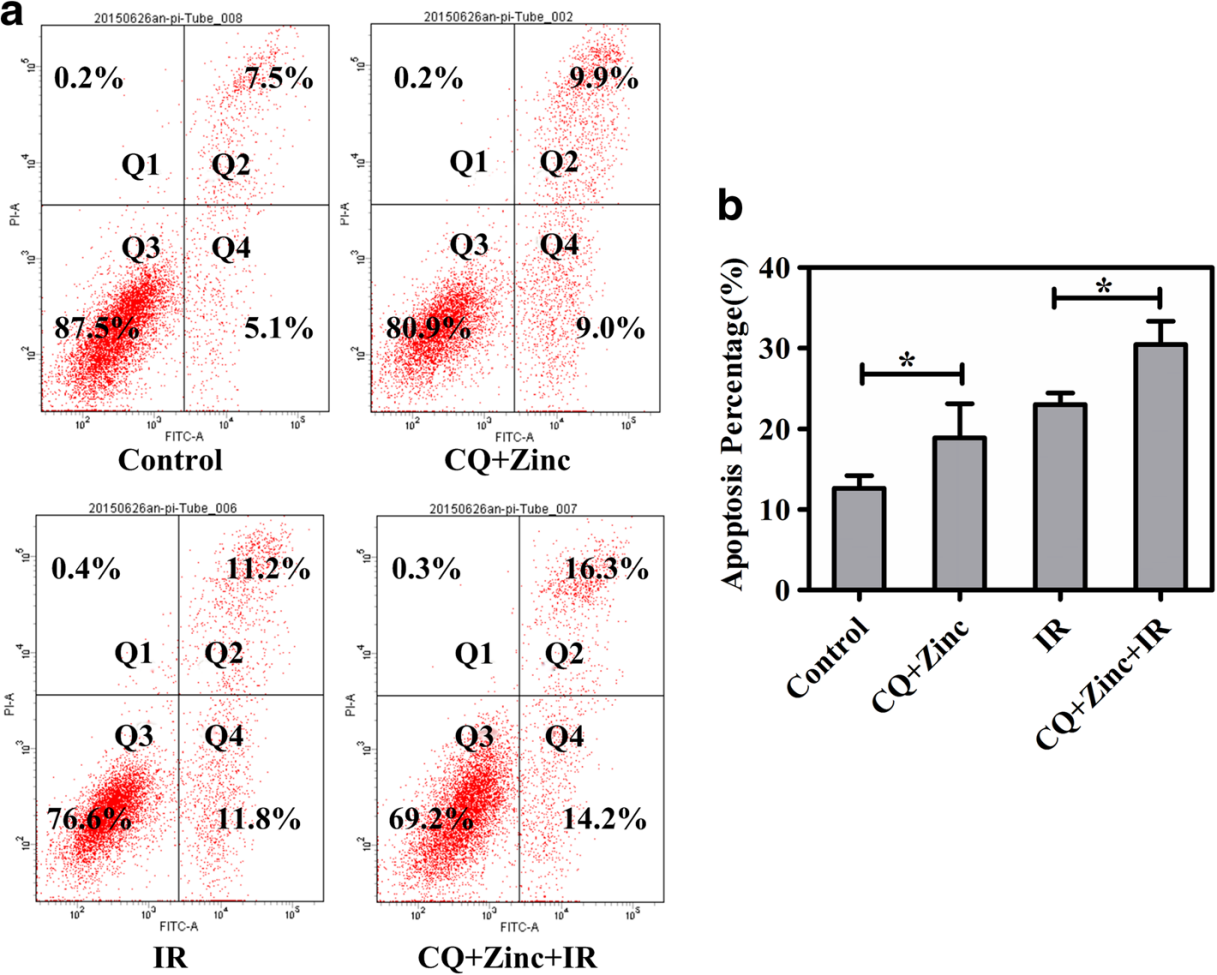
Effects of CQ and zinc on the apoptosis of HeLa cells. **a** and **b**: Cells were treated with 5 μM CQ and 10 μM zinc for 4 h prior to treatment with 6 Gy of irradiation. Apoptosis was measured using propidium iodide (PI)/annexin V double staining in HeLa cells. Representative images of three independent experiments are shown. *P < 0.05

Since reduced clonogenic survival was observed in the clonogenic cell survival assay, we next investigated whether it was resulted from increased apoptosis. As shown in Fig. 3, CQ and zinc treatment enhanced apoptosis in HeLa cells (CQ + Zinc 18.91% vs control 12.64%, *p* < 0.05) and further enhanced the apoptotic response of HeLa cells to 6 Gy of irradiation [30.46% (IR + CQ and zinc) vs 23.04% (IR), *P* < 0.01]. Taken together, these results demonstrated that CQ and zinc enhanced radiation-induced apoptosis in HeLa cells.

### Clioquinol plus zinc combined with γ-ray irradiation modulates the cell cycle distribution

**Fig. 4:**
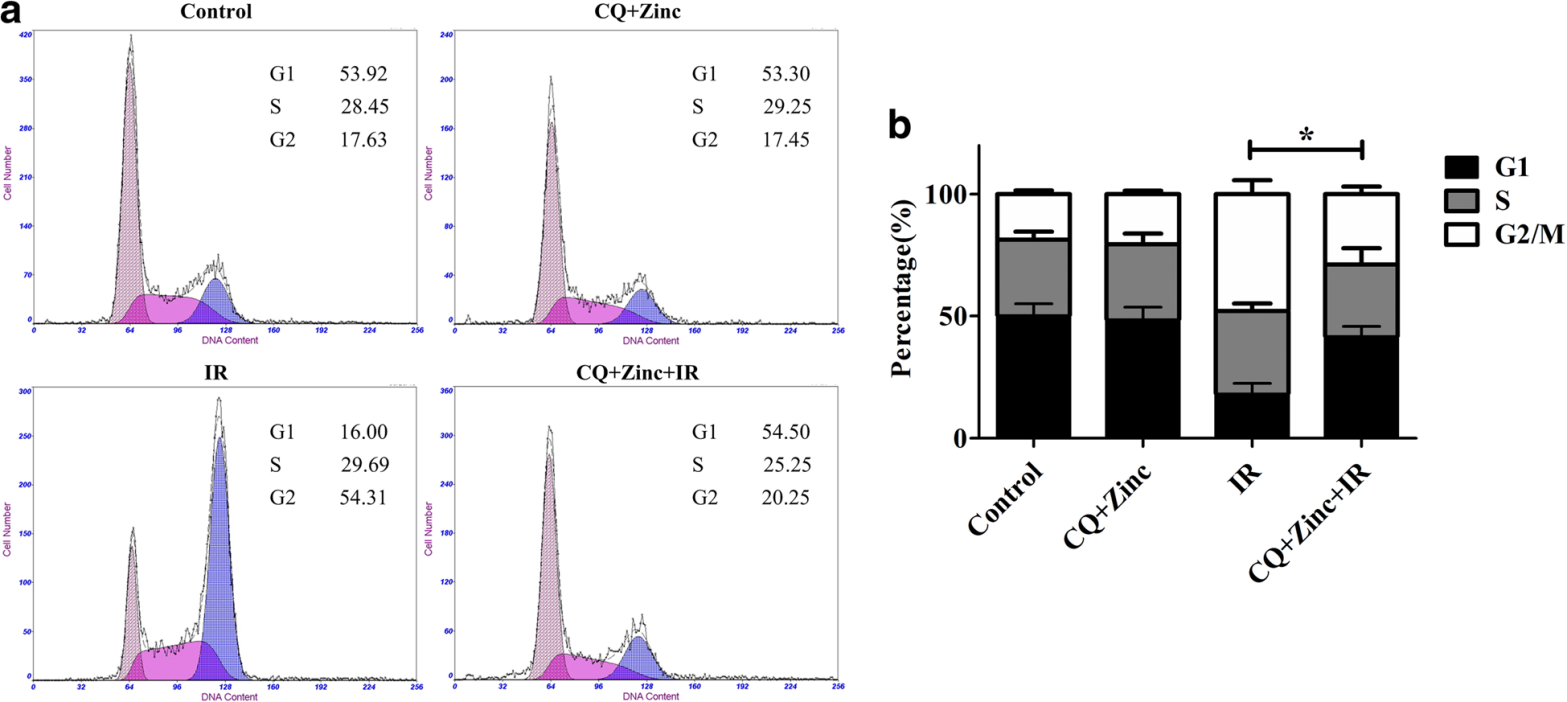
Effects of CQ and zinc on the cycle progression of HeLa cells. **a** and **b**: Cells were treated with or without 5 μM CQ and 10 μM zinc for 4 h prior to exposure to 6 Gy of irradiation (IR). After 24 h, both attached and floating cells were harvested for cell cycle analysis. Shown are representative images of three independent experiments. *P < 0.05

Flow cytometry was conducted to determine whether the CQ and zinc induced radiosensitization was associated with delay in cell cycle. As shown in Fig. 4, radiation induced G2/M arrest in HeLa cells. Compared with untreated cells post-irradiation, cells treated with CQ and zinc plus irradiation showed a decreased population of G2/M arrest in HeLa cells (a reduction of nearly 20%, *P* < 0.05). This result clearly indicated that CQ and zinc partly removed the radiation-induced G2 arrest.

### Clioquinol combined with zinc inhibits NF-κB activity

**Fig. 5:**
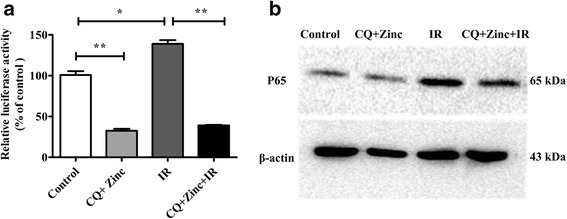
Effects of CQ and zinc on the NF-κB signalling pathway of HeLa cells. **a**: The data were expressed as percentages of the values detected in the control group. ** *p* < 0.01, * *p* < 0.05. Comparison between two groups using independent t test. **b**: Cell lysates were prepared, loaded onto 12% SDS-PAGE gels and blotted with antibodies against p65 and β-actin. Representative images of two independent experiments were shown

To understand whether CQ and zinc inhibit NF-κB activity in HeLa cells, cells were transfected with the pNF-κB-Luc reporter construct and treated with 5 μM clioquinol and 10 μM zinc for 4 h in the presence or absence of 2 Gy of irradiation. Next, we measured the luciferase activity of each group, data are shown in Fig. 5a. Compared with the control group (100%), NF-κB activity was increased to 139% in the radiation group but was decreased to 33% in the CQ and zinc group. Compared with the radiation group (139%), NF-κB activity was decreased to 39% in the CQ plus zinc combined with radiation group. Consistent with this observation, CQ and zinc decreased the total level of nuclear p65, the most frequently detected NF-κB subunit, in the presence or absence of radiation (Fig. 5b). Both of the above findings demonstrated that CQ and zinc down-regulated the NF-κB signalling pathway.

### Clioquinol combined with zinc treatment enhances DNA double-strand-break-induced IR

**Fig. 6:**
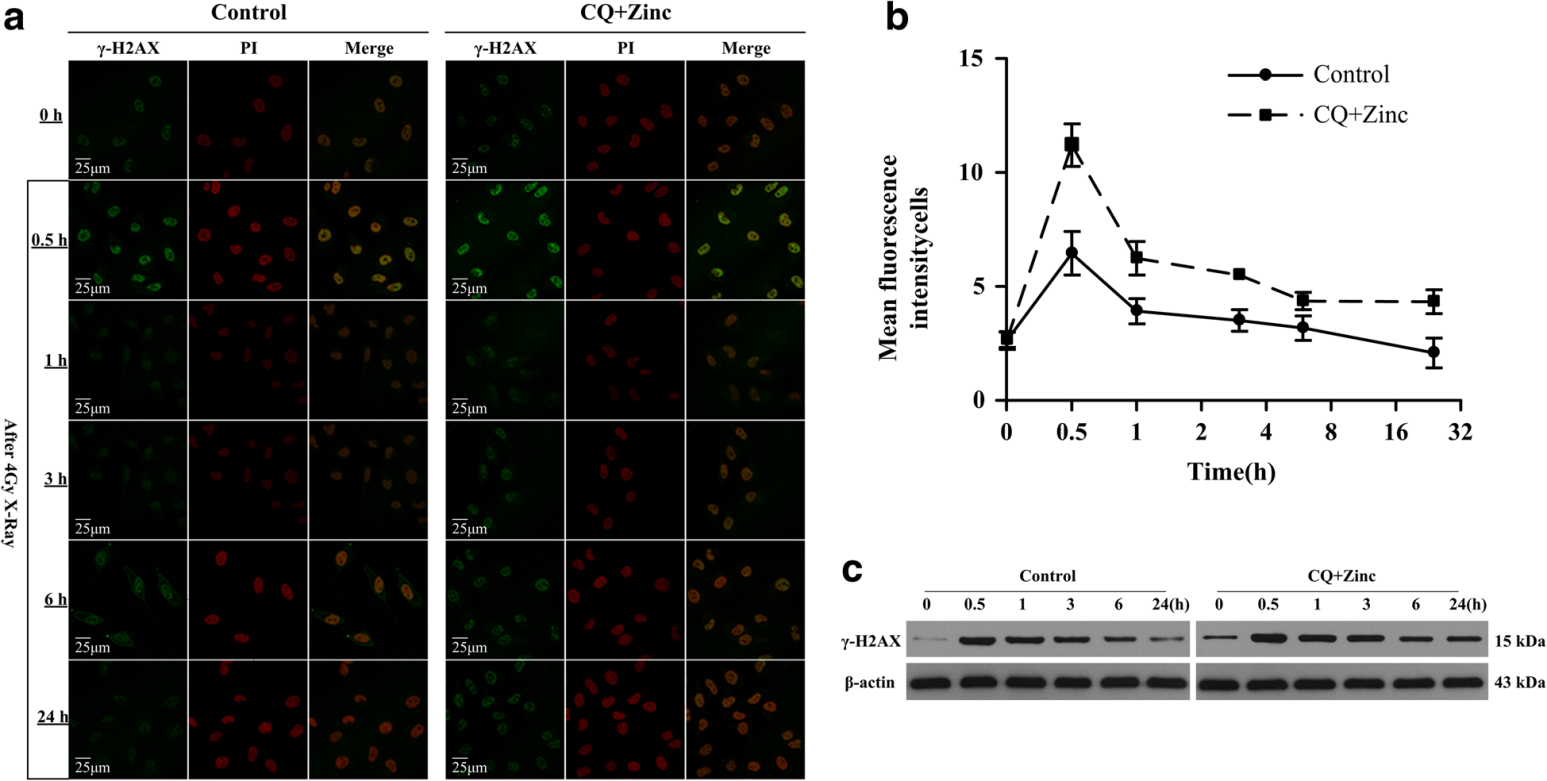
Effects of CQ and zinc on the DNA damage dynamics of HeLa cells. **a** and **b**: Cells were treated with 5 μM CQ and 10 μM zinc in the presence or absence of 4 Gy of irradiation for 0.5–24 h. The cells were stained with propidium iodide (PI) for 10 min and were examined under a confocal microscope. **c**: Cell lysates were prepared, loaded onto 12% SDS PAGE gels (40 μg per well), and blotted with antibodies against γ-H2AX and β-actin. Shown are representative images of two independent experiments

The histone variant H2AX and its phosphorylation on Ser139 (H2AX) is considered a specific DNA double-strand-break (DSB) marker and an important event in the signalling and subsequent repair of DNA DSBs. The protein product of this reaction (γ-H2AX) accumulates at the sites of DSB, forming nuclear foci, where the number of foci is indicative of DNA DSB [13]. We assessed γ-H2AX foci by immunofluorescence as shown in Fig. 6a and b. Cells showed weak fluorescence intensity before irradiation, significantly increasing after irradiation, and especially at 0.5 h. The CQ and zinc group showed a higher mean fluorescence intensity than the control group in various time points and showed significant difference at 0.5 h and 24 h. Additionally, we confirmed this consequence by western blotting (Fig. 6c). There was a significant excess of γ-H2AX expression in the CQ and zinc group compared with that in the control group. Both findings indicated that CQ and zinc could increase radiation-induced DNA DSBs, leading to a delay in the repair process that persisted for 24 h.

### Clioquinol combined with zinc down-regulates the expression of ATM

**Fig. 7:**
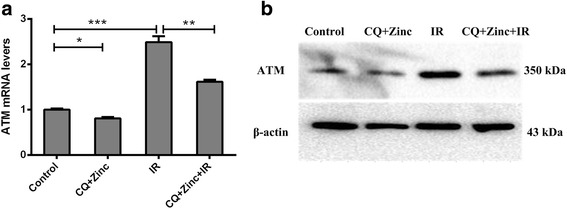
Effects of CQ and zinc on the DNA damage repair of cancer cells. **a** Real-time PCR detected the relative ATM mRNA levels in the CQ + Zinc, Control, IR+ CQ + Zinc and IR groups. **b** Images from western blots to detect the relative ATM protein levels in the CQ + Zinc, Control, IR+ CQ + Zinc and IR groups. Shown are representative images of three independent experiments

ATM is a critical protein of the DNA damage repair pathway, called the DNA damage receptor, and a downstream gene of the NF-κB signalling pathway. Real-time PCR and western blotting were used to analyse ATM expression after 24 h of irradiation. As shown in Fig. 7a and b, CQ and zinc down-regulated the expression of ATM both at the mRNA and protein levels with or without 4 Gy of irradiation. This finding establishes a link between the inhibition of the NF-κB signalling pathway and DNA damage repair signal.

## Discussion

Clioquinol and zinc were reported to induce apoptosis and cytotoxicity in different tumour cells, including prostate, ovarian, hepatoma, pancreatic, and breast cancers, but the effect of clioquinol and zinc on the radiosensitivity of tumour cells has not been fully studied [9]. Here, we found that clioquinol combined with zinc enhanced the sensitivity of cancer cells to ionizing radiation through the inhibition of inherent and radiation-induced NF-κB activity, down-regulation of the DNA damage receptor ATM and increased DSBs, as well as variation of the cell cycle distribution and induced apoptosis.

We previously revealed that clioquinol combined with zinc treatment promoted apoptosis and down-regulated cyclin D1 protein levels in cancer cells [8, 14]. In the present study, we further proved that clioquinol combined with zinc promotes radiation-induced apoptosis and removes G2 arrest. Cell cycle checkpoints of cancer cells is activated when exposed to radiation. However, G1 checkpoint is absent in most cancer cells, commonly resulted from mutations or alterations of key regulators (p53 and Cyclin D1). While activation of the G2 checkpoint, operating primarily via a p53-independent mechanism, is rarely impaired. In fact, disruption of the G2 checkpoint often sensitizes cancer cells without a functional G1 checkpoint to radiation [15, 16]. Taken together, our results indicated that clioquinol combined with zinc treatment increased the radiosensitivity of cancer cells through G2 arrest.

Our previous study found that clioquinol alone targeted NF-κB and lysosome pathways and inhibited NF-κB activity in cancer cells, and the effect became more pronounced in the presence of zinc. Another study reported similar results [17]. In this study, we found that clioquinol combined with zinc significantly inhibited the endogenous and γ radiation-induced activation of NF-κB in HeLa cells. NF-κB signalling is believed to be pro-tumourigenic [18] and usually activated by DNA damage and inflammatory cytokines. It has been found to be in control of multiple cellular processes, including inflammation, transformation, proliferation, angiogenesis, invasion, metastasis, and chemo-radioresistance. Aberrant activation of NF-κB is commonly observed in different cancers [19]. Elevated NF-κB DNA binding activity has been shown in both breast cancer cell lines and primary breast cancer tissues and contributes to malignant progression and chemo-radioresistance and promotes breast tumours metastasis [20]. Moreover, the induction of chemo-radioresistance and anti-apoptotic is mediated by several genes regulated by NF-κB, and inhibition of this transcription factor increases the sensitivity of cancer cells to chemotherapeutic agents and radiation [21–24].

Thus, the inhibition of NF-κB represents a promising therapeutic strategy, and transient radiation-inducible NF-κB activation presents a pro-survival response to radiation that may account for the development of radioresistance [25]. Our results suggested that clioquinol combined with zinc promotes the radiosensitization of HeLa cells and potentiates the antitumour effects of radiotherapy by blocking this signalling pathway.

It is believed that DNA double-strand breaks are the crucial cytotoxic lesion from ionizing radiation, and DNA damage and repair ability is critical for tumour cells to survive from radiation. The major methods for DSBs reparation are non-homologous end joining (NHEJ), single-strand annealing (SSA) and homologous recombination (HR). NHEJ is observed throughout cell cycle, while HR is limited to S/G2-phase of cell cycle [26]. To investigate the radiosensitizing mechanism of clioquinol combined with zinc, we evaluated DSBs damage and repair by quantifying γH2AX foci, the phosphorylated form of the variant histone H2AX, which is a marker of DSBs damage [27]. In our research, we demonstrated that clioquinol combined with zinc increased the formation of γ-ray irradiation induced γ-H2AX foci on both macro and micro levels and delayed the degradation of γ-H2AX foci, apparent within 0.5 h of irradiation and sustained for 24 h. Meanwhile, we found that clioquinol combined with zinc down-regulated the expression of ATM, which played a prominent role in DNA damage and repair. ATM is considered to be a primary transducer of cellular responses to DSBs and activate various damage response signalling pathways, including cell cycle checkpoints, DNA repair, and the induction of apoptosis, explaining why the ratio of G2 arrest declined to some extent. Although NF-κB target genes, encoding DSB repair factors, were identified, little is known about the functional role of NF-κB in DSB repair. It was previously shown that loss of p65 in murine fibroblasts compromised DNA repair and genome stability during cellular senescence [28]. However, contradictory conclusions were drawn in glioblastoma cells, where NF-κB promotes the accumulation of ssDNA breaks and apoptosis [29]. Recently, it was revealed that NF-κB transcriptionally up-regulates ATM, and higher ATM kinase activity assures fully efficient HR through downstream signalling cascades, thus strongly stimulating the removal of DSBs [30].

Our findings established a link between the inhibition of the NF-κB signalling pathway and the DNA damage and repair process. We deduced that clioquinol combined with zinc inhibited NF-κB activity, leading to down-regulation of ATM, and deficient HR to remove DSBs.

## Conclusions

As far as we know, the present research is the first to report that clioquinol combined with zinc could increase the radiosensitivity of cancer cells at a low toxic concentration. The putative mechanisms include the abolishment of G2 cell cycle arrest, enhancement of apoptosis, inhibition of the NF-κB-ATM pathway, induction of more DNA damage and inhibition of the repair of RT-induced DSBs damage. Our current findings support the notion that clioquinol combined with zinc is expected to be a promising treatment modality for cancer radiotherapy (RT), and we are planning on validating the effects of clioquinol combined with zinc in xenograft animal models.

## Abbreviations

CCK-8: Cell counting kit 8
CQ: 5-chloro-7-iodo-8-hydroxyquinoline
DMSO: Dimethyl sulfoxide
DSBs: Double-strand breaks
HR: Homologous recombination
NHEJ: Non-homologous end joining
RT: Cancer radiotherapy
SER: Sensitizer enhancement ratio
SSA: Single-strand annealing
SSD: Source skin distance
